# Autogeny in *Culiseta longiareolata* (Culicidae: Diptera) mosquitoes in laboratory conditions in Iran

**DOI:** 10.1186/s13104-020-04942-5

**Published:** 2020-02-19

**Authors:** Fereshteh Ghahvechi Khaligh, Abdollah Naghian, Shadiyeh Soltanbeiglou, Saber Gholizadeh

**Affiliations:** 1 Cellular and Molecular Research Center, Cellular and Molecular Medicine Institute, Urmia University of Medical Sciences, Urmia, Iran; 2 Medical Entomology Department, School of Public Health, Urmia University of Medical Sciences, Urmia, Iran

**Keywords:** Culicidae, *Culiseta longiareolata*, Autogeny, Iran

## Abstract

**Objectives:**

*Culiseta longiareolata* is a cosmopolitan species and has implicated in the transmission of avian malaria, tularemia, and arboviruses. Despite the wide distribution of *Cs. longiareolata* in Iran, little is known about its biology and physiology. The current research was conducted to study the autogeny behavior in this potential vector. During 2018, larvae and pupae were collected from Nazloo region in Urmia City using standard methods. Mosquitoes were reared in cages and fed by 5% sugar in laboratory conditions and were then dissected in phosphate-buffered saline (PBS) under a stereo microscope.

**Results:**

In total, 230 adult female *Cs. longiareolata* mosquitoes were dissected. Egg rafts were observed in the ovary of only 10.86% unfed female mosquitoes. Autogeny behavior is a significant factor in the growth of population without a blood feeding. Therefore, it is necessary to study how autogenous reproduction affects mosquito-borne diseases.

## Introduction

Culicidae family is comprised of 113 genera and 3570 species [1] with a broad distribution throughout the world [2]. In 1878, mosquitoes were the first arthropods officially introduced as the intermediate hosts of vertebrate parasites; however, they are now recognized as the most important arthropods affecting human health [3]. Indeed, mosquitoes are the vector of various diseases such as Malaria, Filariasis, Yellow Fever, West Nile fever, Japanese Encephalitis, Rift Valley fever, etc. [4–7].

Typically, the development of eggs in blood-sucking insects is normally dependent on a blood meal [8]; nonetheless, there are some female mosquitos that can develop their eggs without a blood meal [9], a trait called autogeny [8, 10]. In a number of females of this group of insects, the first group of eggs and possibly the next can grow without blood feeding [11, 12]. For the growth, they often use the last larval stage food or feed on sugared salmon. This characteristic has been observed in many species of mosquitoes [8]. Autogeny is necessary for some insect species such as Megarhinini because their semiflexible proboscis is specialized for nourishment from plants [11].

*Culiseta longiareolata* is a species of the family Culicidae, the Culicinae subfamily [13] and a vector of avian malaria [14], tularemia [15], and arboviruses such as West Nile fever [16–18]. This multivoltine, thermophilic, and ornithophilic species is distributed in Europe, Asia, and Africa, as well as in the Mediterranean Sea [19]. It mainly develops in small water bodies, and the adults may enter houses and attack humans [20], though their primary hosts are birds [15]. This mosquito species are readily distinguished from other *Culiseta* species [21], and its morphological characters include white stripes and points on legs, head, and thorax [22].

The purpose of this research was to investigate the autogenous reproduction in *Cs. longiareolata.* This result might be valuable in the survival of the vector species in the absence of a host and the vectorial capacity. Owing to the uncertainty about the influence of autogeny on the vector role of mosquito, it is essential to assess the basis of this autogeny.

## Main text

### Methods

**Fig. 1:**
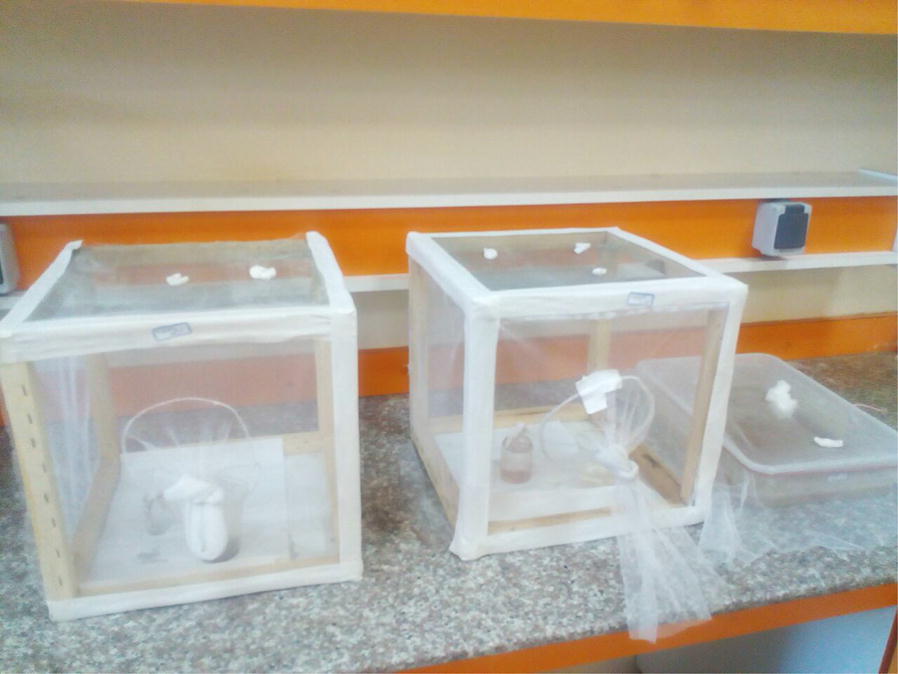
Field-collected *Cs. longiareolata* mosquitos reared in laboratary conditions

Urmia, the largest city of West Azerbaijan Province, is located on a 70-mile-wide plain in a 18-km distance from Lake Urmia and situated in the latitudes of 44° 52″ 35″ S and 37° 39′ 13″ N. During the spring and summer in 2018, larvae and pupae were collected from Nazloo region (37°31′ 59.2″ N 45°02′ 54.5″ E) in Urmia by using standard dipping techniques [23]. The collector’s name, global positioning system (GPS) coordinates of the area positioning, and date of capture were recorded. Following the sample collection of mosquitos with different larval stages from their habitats, they were transferred to the medical entomology laboratory in the Urmia University of Medical Sciences, School of Public Health (Urmia, Iran) for rearing. The laboratory temperature was set at 22–25 ℃, and room lighting was provided with a yellow light. The samples were kept for 10 h in the darkness and 14 h in the brightness. A plastic cuvette-like container with the dimensions of 45 × 25 × 5 cm was used to maintain immature stages (eggs, larvae, and pupes). More than half of the container was filled with the larval habitat water and kept in 22–25 ℃; approximately 200 larvae were placed in the container. After 2–3 days, the pupae were taken with a plastic sucker and placed in disposable glasses in cages (30 × 50 × 80 cm) until the emergence of adult mosquitoes (Fig. 1); the cages were covered with finely textured cloth. Adults were feed on a cotton impregnated with 5% sugar. The cotton was streaked with a glucose solution daily and was replaced with a new sugar solution every 3 days to prevent the growth of fungi. Pupae were identified to species level using the morphological key [24]. The newly emerged males and females of *Cs. longiareolata* were then transferred to new cages for mating and dissecting in PBS, to obtain eggs under a stereo microscope. The test was repeated three times.

### Results

**Fig. 2:**
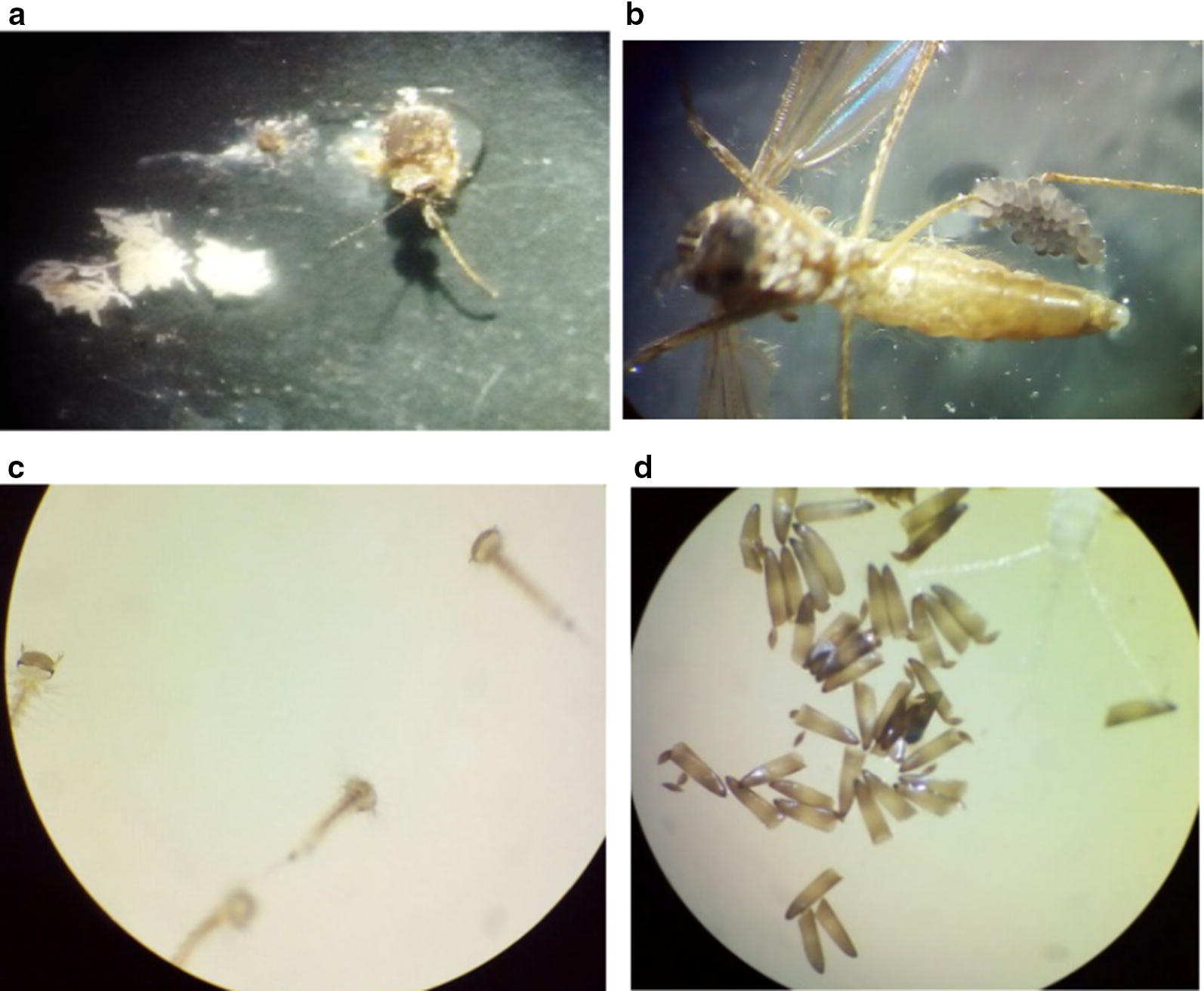
**a** Eggs in the ovary of dissected autogenous *Cs. longiareolata* mosquitoes; **b** eggs laied by autogenous *Cs. longiareolata* mosquito; **c** egg shells **d** the first larval instar of *Cs. longiareolata* emerged from autogenous mosquitoes

A total of 230 adult female *Cs. longiareolata* mosquitoes were used in the mating experiments in three replications (Fig. 2a). Within 15–21 days after emerging, the abdomen of mosquitoes enlarged. Each gravid mosquito was incubated in a separate cage for egg laying. Three days after incubation, eggs were laid on a cup of water (Fig. 2b) and transferred to new plastic containers. The color of eggs was grey immediately after laying, but 1–2 day(s) later, the color was changed to black or dark brown. The first instar of the larvae was emerged within 3–5 days (Fig. 2c, d). Of all the female adults, 10.86% produced eggs without a blood feeding. Interestingly, autogenous females of *Cs. longiareolata* had larger body size than anautogenous specimens.

### Discussions

There are numerous investigations related to Culicidae identification, distribution, insecticide resistance, and vector control in Iran [4, 7, 25–30]. *Anopheles sacharovi* experiences gonotrophic dissociation during autumn and winter in Northern Iran [28]. Anautogeny has been reported in *Culex pipiens* in the central parts of the country [31], but there are no report on the autogeny in *Cs*. *longiareolata* in Iran. Therefore, the current study, for the first time, reports autogeny in *Cs*. *longiareolata* in the country.

Autogenous species have higher nutritional requirements than anautogenous species, in order to develop their egg batch [32]. Temperature, environmental factors, geographical variation [33], seasonality [34], and food availability [35] help mosquitoes become autogenous. For instance, when larvae fed on an animal matter is more likely to be autogenous and to produce more eggs in *Cs*. *longiareolata* because of the high protein content of their food [36]. Besides, very few populations of *Cs. longiareolata* are autogenous, and all the laboratory mosquito populations are anautogenous [37, 38]. In the present study, the autogeny rate was low (10.86%) in *Cs*. *Longiareolata* collected from Urmia, and larvae were gathered from a larval habitat near livestock and reared in water without any other nutrient additive. Our results regarding the larger body size of autogenous *Cs. longiareolata* mosquitoes confirmed that the growth of body size in Culicidae is a general phenomenon [39, 40]. However, relationship between the enhancement of the fertility and pupal mass and wing length has been documented in *Aedes albopictus* and *Aedes geniculatus* mosquitoes [41]. Perhaps the larval nutrition is a significant factor in autogeny in *Cs. longiareolata* in Northwestern Iran, and the determination of physicochemical characters of larval habitat water may contribute to better understanding of *Cs. longiareolata* autogeny.

It is hypothesized that autogeny can affect different parameters of pathogen transmission, such as increasing the vector population and reducing the pathogen incubation period [42]. Therefore, autogeny is an excellent survival mechanism for a particular mosquito species [9, 42, 43]. The present study showed a more extended survival period in females fed on a 5% sugar solution. The high survival rate enhances the ability of mosquitoes to transmit possible pathogens. However, autogenous species could transmit arboviruse pathogens more horizontally than anautogenous populations [32].

### Conclusion

*Cs. longiaerolata* is one of the potential vectors of a number of vector-borne diseases such as avian malaria, bird flu, avian smallpox, and Malta fever. Urmia city is an excellent destination for many migratory birds because of its lakes and islands. The presence of migratory birds as the reservoir and *Cs. longiaerolata* as potential vector of infectious disease provide conditions for transmitting infectious diseases. However, their role of autogeny on transferring infectious diseases needs to be studied.

## Limitations

The major concern of this study is the examination of autogeny on specimens emerged from the field-collected larvae. In virtue of our limitations in insectary, we could not follow autogeny in the next generation. The current study was conducted only on specimens collected from the Nazloo region in Urmia district. The test of autogeny on samples from various regions and in large areas in the country is recommended.

## Data Availability

Data supporting this article are included in the article.

## Abbreviations

PBS: Phosphate-buffered saline
GPS: Global positioning system
